# Seismic Detection of a Magma Reservoir beneath Turtle Island of Taiwan by S-Wave Shadows and Reflections

**DOI:** 10.1038/s41598-018-34596-0

**Published:** 2018-11-06

**Authors:** Cheng-Horng Lin, Ya-Chuan Lai, Min-Hung Shih, Hsin-Chieh Pu, Shiann-Jong Lee

**Affiliations:** 1 Institute of Earth Sciences, Academia Sinica, Taipei, Taiwan; 2 National Center for Research on Earthquake Engineering, National Applied Research Laboratories, Taipei, Taiwan; 3 Taiwan Volcano Observatory at Tatun, Taipei, Taiwan; 4 Department of Geosciences, National Taiwan University, Taipei, Taiwan; 5 Central Weather Bureau, Taipei, Taiwan

## Abstract

Although surface geology, eruption information and clustering seismicity all suggest Turtle Island (Kueishantao) of northern Taiwan is an active volcano, there was no direct evidence to conclude that magma reservoirs exist beneath it. Even less evidence is available to determine their spatial configuration. If the magma reservoirs are filled by liquids and melt, S-waves are totally reflected and leave behind a shadow, like when passing through the Earth’s outer core. We detect both these S-wave shadows and strong reflections from the surface using earthquakes at different depths and azimuths. These observations identify a km-scale molten-filled volume located beneath Turtle Island. The magmatic nature of the reservoir is supported by the onset of non-double-couple earthquakes with strong CLVD (Compensated Linear Vector Dipole) and ISO (Isotropic) components, which show a tensor crack compatible with some volume changes within the reservoir. Combining these results with two independent 3-D velocity models and aeromagnetic anomalies recorded in Taiwan, a partially-molten ~19% low-velocity volume is estimated in the mid-crust (13–23 km), with spatial uncertainties of ~3 km. The elongated direction approximately follows the strike of the Okinawa trough, indicating that the source of the magma reservoir might be a back-arc opening.

## Introduction

Since magmatic eruptions are the result of hot and liquid magma ascending from the subsurface to volcanoes, a magma reservoir in the crust is often observed as the source for supplying magma. However, there is still debate about what a magma reservoir is exactly^1,2^. To improve the understanding of the spatial distribution of molten magmas within a reservoir, seismic images often play one of the most important roles. The general geometries of magma reservoirs have been successfully delineated either from seismic low-velocity^3–5^ or anisotropy zones^6^, but those seismic images only reflect the general characteristics of the partial melting rocks in the magma reservoir. Until recently, there was no direct seismic evidence to detect liquid magma within the reservoir. Some molten magmas were detected within the reservoir beneath the Tatun volcano group of Taiwan based on the consistent evidence of both S-wave shadows and P-wave delay^7^. The results further suggested that the magma reservoir was not completely dominated by molten magma, but it was probably filled by either a number of melt sills or a thin magma layer on the top only.

To further determine which model is more appropriate for describing a magma reservoir, in this study we check not only S-wave shadows but also strong S-wave reflections because S-waves could not propagate into the liquid body^8^. At first, we examine S-wave shadows from several felt earthquakes (M > 4) at different depths around an active volcano (Turtle Island) offshore northeast Taiwan. Similar observations of the S-wave shadow were reported in detecting magma reservoirs in Krafla Caldera in northeastern Iceland^9^ and the Tatun volcano group in northern Taiwan^7^. In addition to the nearly vertical ray-paths from the upper mantle to the surface, we carefully cross-check the horizontal ray-paths within the crust to reveal the physical state inside a magma reservoir. Then, the location of the partial melt-rich reservoir obtained by S-wave shadows is further confirmed by the strong S-waves reflected from the partial melt-rich reservoir. Although the P-wave seismic reflections have been employed to detect magma reservoirs in several places^10–12^, we focus on the S-wave reflections that might be more sensitive to the melt-rich reservoirs. Both S-wave shadows and strong reflections are simulated using some simplified 2-D structures to estimate the suitable location of the magma reservoir. In order to further support the interpretation of magma chamber beneath Turtle Island, we found two representative non-double-couple earthquakes with strong CLVD (Compensated Linear Vector Dipole) and ISO (Isotropic) components for showing the tensor crack with some volume changes within the magma chamber. Combining those results with the previous results of seismic tomography and airborne magnetic survey, then a volcanic complex of partial melt-rich sills and dikes is proposed for describing the magma reservoir beneath Turtle Island of Taiwan.

## Turtle Island

**Figure 1:**
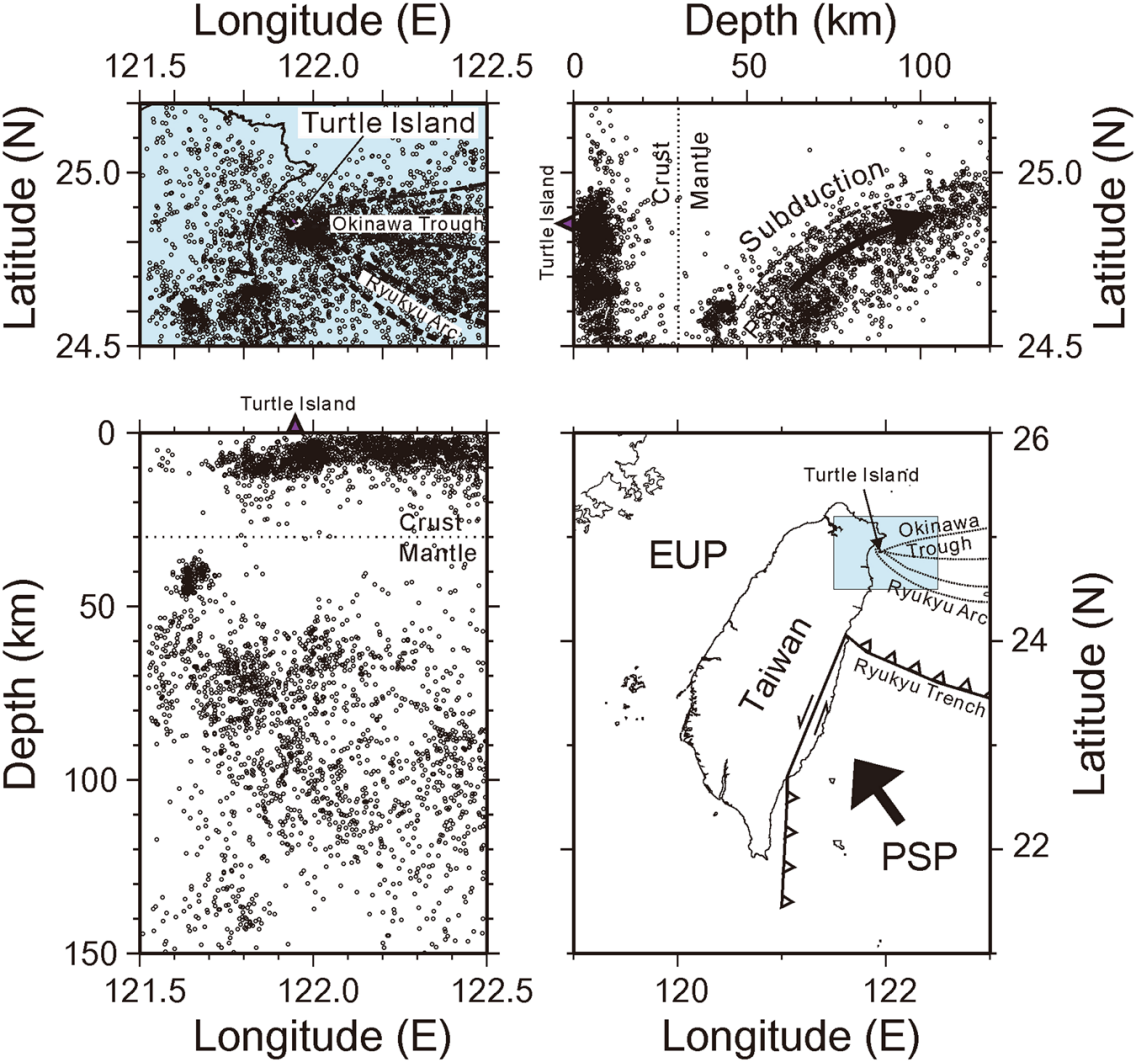
Seismicity and general tectonics in the NE Taiwan area, which is located at the westernmost end of the Okinawa trough. Background seismicity is divided into two groups. The shallow earthquakes in the upper crust are associated with the opening of the back-arc basin along the Okinawa trough, and the deep earthquakes are located within the Benioff zone of the subducted PSP (Philippine Sea Plate).

Turtle Island (or Kueishantao), named for its shape, is located offshore the Ilan plain in the northeastern Taiwan area (Fig. 1). From the tectonic point of view, Turtle Island is not only situated at the westernmost end of the Okinawa trough but also along the Ryukyu volcanic arc. In addition to the Tatun volcano group^7,13–15^, Turtle Island has been identified as one of the active volcanoes in the Taiwan area^16,17^. This volcanic island is probably associated with the back-arc opening along the Okinawa trough as the Philippine Sea plate subducts beneath the Eurasian plate along the Ryukyu trench between Taiwan and Japan^18–21^. Consequently, many earthquakes have been detected in and around Turtle Island (Fig. 1) and routinely reported by the Central Weather Bureau in Taiwan^22^. Those background earthquakes are clearly divided into two groups according to their focal depth variations. Shallow earthquakes, with depths less than 15 km, are largely associated with the opening of the back-arc basin along the Okinawa trough and with volcanic activity^23–25^. For instance, a low-frequency, large non-double-couple earthquake was observed to demonstrate fluid involvement at its source^26^ because similar non-double-couple earthquakes were detected near the well^27^ or by hydraulic injections^28^. For deep earthquakes, with depths greater than 50 km, a clear Benioff zone is delineated^29^. The subducted slab is identified about 100 km beneath Turtle Island, which is a typical location for the volcanic arc along the Ryukyu subduction system^20,21^.

Although Turtle Island is offshore northeastern Taiwan, the impact of possible hazards cannot be ignored (complex hazards might be considered) if the volcano erupts again. Some volcanic activities such as lava flows were shown by historical accounts in 1775–1795, but the geological dating results of siltstone xenolith show the last eruptions was later than 7 ka^16^. Turtle Island is part of Caldera and the major part of the volcanic cone is covered by the sea water. In addition to direct volcanic impacts, such as lava flow and volcanic ash, there is the potential to generate a tsunami due to the volcanic collapse causing the southern flank of Turtle Island to slide into the ocean. Although there was no direct record to show any tsunami caused by the large collapse, some small scale of collapses at steep cliff were occasionally induced by strong earthquakes (Fig. A1). Also, a concave edifice at the northeastern flank was probably created by a landslide^17^. Thus, the Ilan Plain, which has about a half-million residents, might be seriously damaged by the tsunami waves due to almost half of the plain having an extremely low altitude (<10 m). A similar case being considered is a future collapse by eruption or others at La Palma, Canary Islands offshore of western Africa, in which tsunami generated by the 500 km^3^ slide block could transit the entire Atlantic Basin and arrive at the eastern coast of the Americas with a height of 10–25 m^30^. Although the possibility of such a future hazard is still under debated, one of the worst cases in the history was the 1792 Unzen volcano eruption^31^, which caused ~15,000 fatalities (mainly by tsunami). Therefore, to help understand the possible hazards we have collected and analyzed seismic, geochemical, and geophysical data for Ilan Plain and Turtle Island for monitoring any possible volcanic activity.

**Figure 2:**
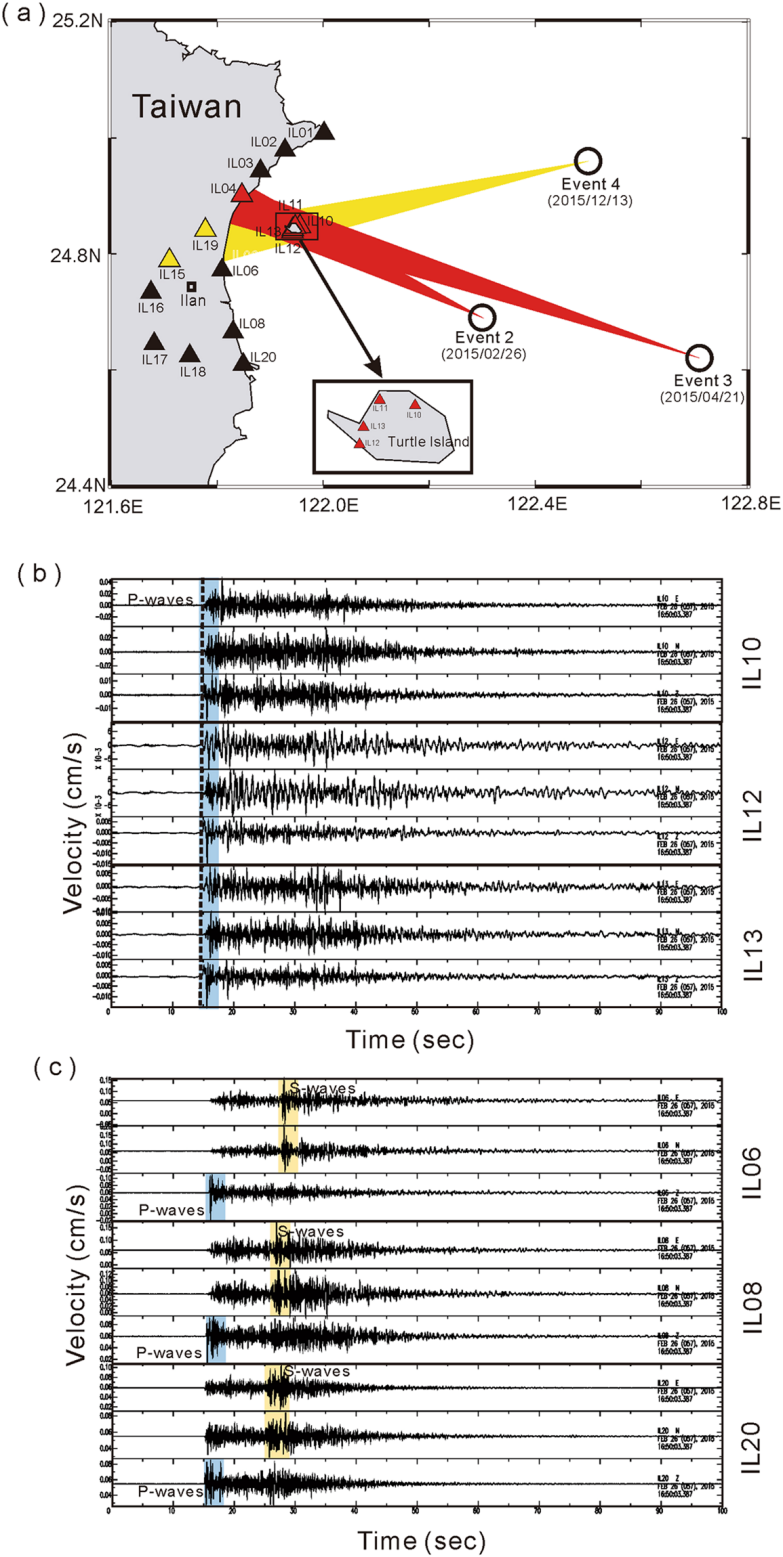
Cross-check of S-wave shadows in the NE Taiwan area. (**a**) Locations of seismic stations (triangles) and 3 earthquakes (circles). The color beams (red and yellow) from earthquakes to seismic stations indicate the ray-paths in which S-wave shadows are detected. Comparison between 3-component velocity seismograms recorded at seismic stations (**b**) without and (**c**) with S-waves from Event 2.

**Figure 3:**
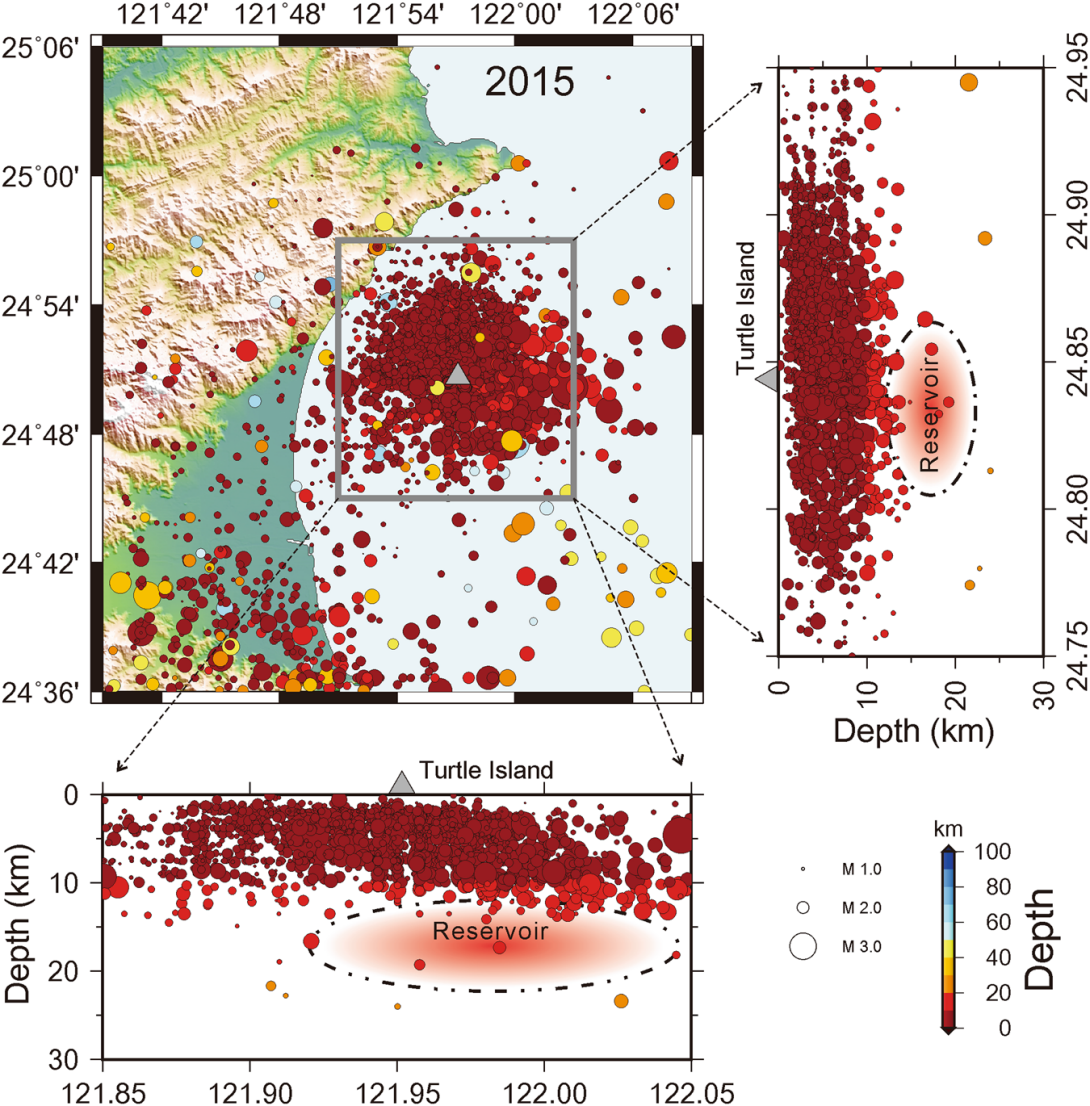
Background seismicity in and around Turtle Island in 2015. Colorful circles with different sizes show the distributions of earthquake depths and magnitudes, respectively. The magma reservoir in red is roughly delineated by a broken-line circle.

Although surface geology, eruption information and clustering seismicity mentioned above suggest Turtle Island is an active volcano, there was still no direct evidence to conclude whether magma reservoirs exist beneath it or not. In order to improve the ability of detecting volcano-earthquakes for understanding possible volcanism in and around Turtle Island, in 2008 we installed a seismic network in the Ilan county of Northeastern Taiwan (Fig. 2a). The seismic network includes 12 seismic stations in Ilan Plain and 4 others on Turtle Island^28^. Although the deployment of some OBS (Ocean Bottom Seismometers) might be helpful, it is prohibited by frequent fishing activity because Kuroshio oceanic current just passes through Turtle Island. Each seismic station is equipped by either a three-component Guralp 6TD or H802 sensor with a sampling rate of 100 samples/second. Seismic data recorded at most seismic stations, including those at Turtle Island, are transmitted in real-time to the Taiwan Volcano Observatory at Tatun (TVO) and the Institute of Earth Sciences, Academia Sinica to monitor for any possible volcanic activity in and around the island. Figure 3 shows crustal seismicity detected in and around Turtle Island in 2015. It is worth mentioning that most of the earthquakes were clustering around the island with the depths of less than 15 km, while there are very limited earthquakes away from Turtle Island. Such a clustering seismicity in and around Turtle Island, like that in the Tatun volcano group^7^, might be likely associated with a possible magma reservoir.

**Figure 4:**
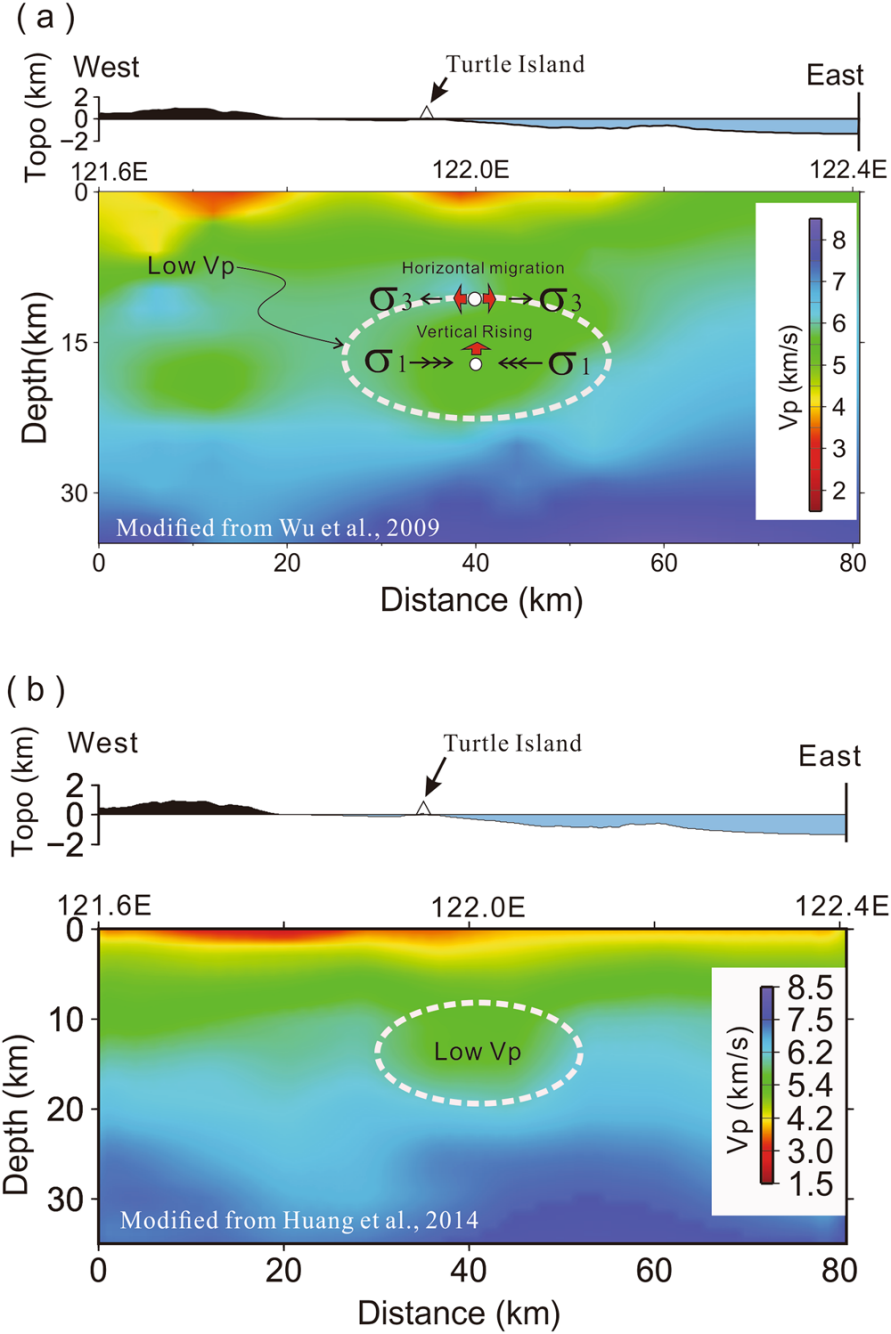
P-wave velocity profiles across Turtle Island (Latitude = 24.85°N) modified from (**a**) Wu *et al*., 2009 and (**b**) Huang *et al*., 2014. A low velocity zone (LVZ, dashed-circle) is consistently obtained from both tomographic images. Two CLVD earthquakes (white circles shown at the upper panel) indicate the vertical rising might be resulting from the horizontal compression (σ1: maximum stress) within LVZ while the horizontal migration was associated with the horizontal extension (σ3: maximum stress) around its top.

In fact, the general geometry of a possible magma reservoir shown at Fig. 3 is highly comparable to the low velocity zones (LVZs) obtained from two independent seismic results of 3D tomographic inversions by using different data set and methods^32,33^. To focus on northeastern Taiwan^32^, seismic data recorded in Taiwan as well as Okinawa Islands of Japan were first combined together for tomographic inversion in 2009. In total, they employed 19,143 earthquakes recorded by TSMIP (Taiwan Strong Motion Instrumentation Program), CWBSN networks (Central Weather Bureau Seismic Network), JMA (Japan Meteorological Agency) and some OBSs (Ocean Bottom Seismometers). Later on a new result in 2014 employed more seismic data for doing tomographic inversion^33^. In addition to P- and S-wave travel times recorded in Taiwan and Japan, for instance, the borehole logging data were added for correcting near-surface structures in the whole Taiwan area. Totally, almost one million readings of P-wave, S-wave and S-P times were selected from 69,353 earthquakes recorded at 1,112 stations. The comparison between both tomographic images across Turtle Island shows a LVZ with some slight difference in size is consistently obtained around the depths between 10~20 km (Fig. 4). Although both resolutions might be not very high offshore northeastern Taiwan, the general pattern of the LVZ might be reliable. It is very likely to suggest a possible magma reservoir beneath Turtle Island.

## S-wave Shadow

Careful examination of seismic data generated by the deeper earthquakes (focal depth >90 km) within the subduction zone in the northeastern Taiwan area shows some interesting results for identifying a partially molten volume in the crust. We examined more than 40 deeper earthquakes occurred offshore northeastern Taiwan in 2014–2015, and found some of them were lack of S-waves or strong scattering at Turtle Island (Fig. A2). It is well known that both P- and S-waves are often identified in the seismograms generated by a local deep earthquake since the mantle is significantly less heterogeneous than the crust. For instance, the 3-component seismograms generated by a local earthquake at a depth of 93.2 km just beneath Ilan Plain (Event 1 in Table 1) and recorded at Station IL10 on Turtle Island shows clear P- and S-waves (Fig. A3). Surprisingly, it is hard to identify S-waves from the seismic data recorded at the same station generated by another deep earthquake at a depth of 97.6 km southeast to Turtle Island (see Fig. A2 and Event 2 in Table 1). Based on the CMT (Centroid Moment Tensor) focal mechanism inverted by seismic data recorded at the Broadband Array in Taiwan for Seismology (BATS)^34^, the incidence ray at Station IL10 is not coming from the exact direction in which S-waves are absent (Fig. A3). Thus, the missing S-waves are called shadows along the ray-path between the earthquake and seismic station. But such shadows cannot be caused by any liquid or strong heterogeneous volume just beneath the seismic station (IL10) because Events 1 and 2 share nearly identical paths within the uppermost crust.

**Table 1:** Earthquake parameters (Provided by Central Weather Bureau in Taiwan).

Earthquakes	Longitude	Latitude	Depth	Mag. (M_L_)	Year/Mo/Dy	Hr/Min
Event 1	121.82°E	24.76°N	93.2 km	4.55	2015/03/11	23/02
Event 2	122.30°E	24.69°N	97.6 km	5.25	2015/02/26	14/50
Event 3	122.71°E	24.62°N	20.0 km	4.28	2015/04/21	09/51
Event 4	122.50°E	24.96°N	16.9 km	4.00	2015/12/13	06/50
Event 5	122.02°E	24.87°N	11.9 km	4.58	2015/09/02	02/18
Event 6	121.98°E	24.83°N	9.3 km	4.02	2015/10/19	07/26
Event 7	121.99°E	24.84°N	6.2 km	4.07	2015/10/19	07/31
Event 8	121.98°E	24.85°N	5.6 km	4.00	2015/10/20	23/08
Event 9	122.03°E	24.85°N	8.9 km	4.77	2015/11/03	00/06
Event 10	121.91°E	24.39°N	42.6 km	4.31	2015/05/24	08/35
Event 11	122.01°E	24.83°N	16.0 km	3.7 (Mw)	2016/11/29	06/39
Event 12	122.01°E	24.87°N	8.0 km	4.8 (Mw)	2015/11/03	00/06

**Figure 5:**
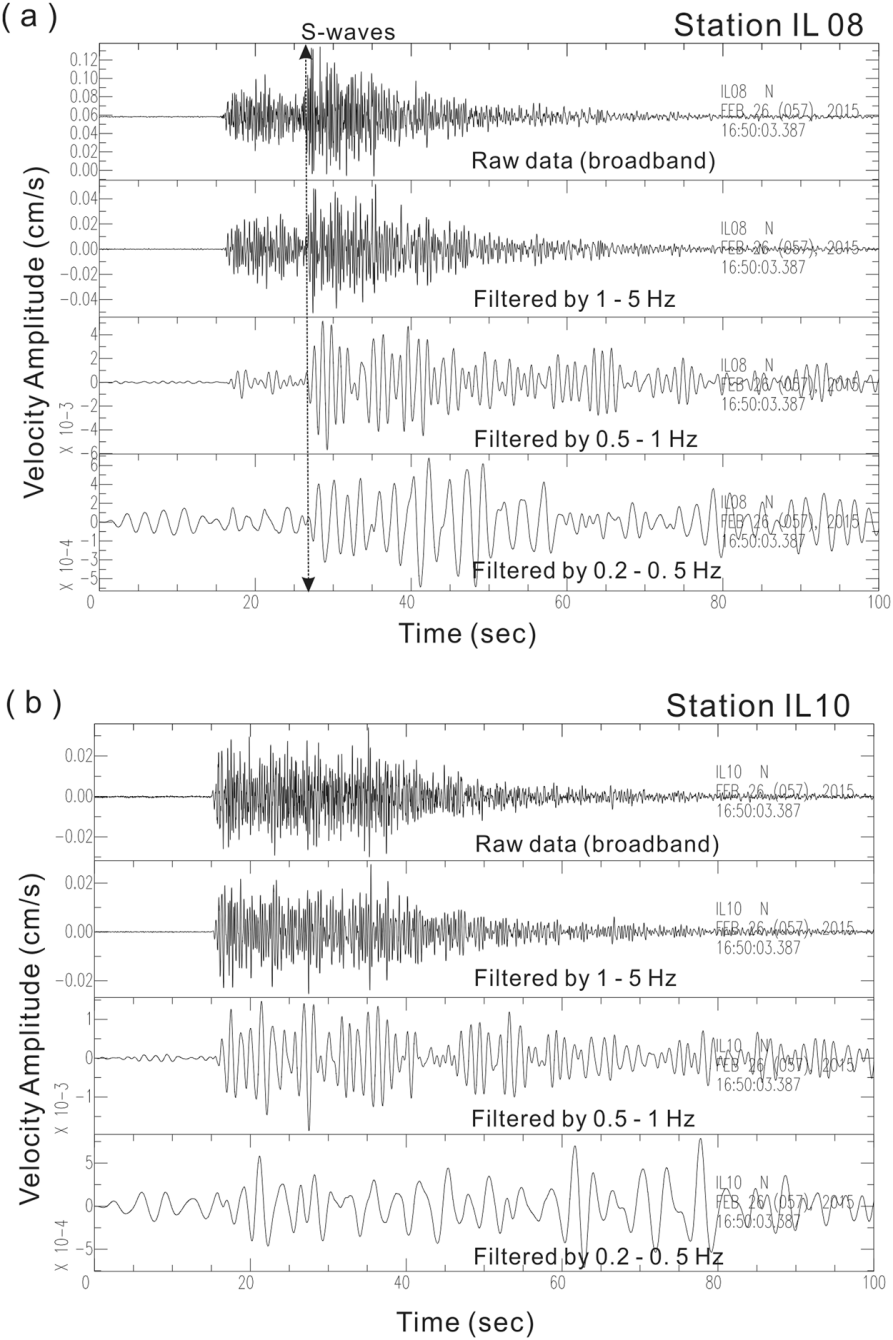
Comparison of S-wave arrivals between two seismic stations (IL10 and IL08). (**a**) S-wave arrivals at all of different frequency bands were clearly identified at IL08, while (**b**) they were hardly observed at IL10.

In fact, the S-wave shadow generated by Event 2 is not only found at station IL10, but also at other seismic stations (IL11 and IL12) on Turtle Island and another station (IL04) along the coast of Ilan Plain (Fig. 2b). In stark contrast, clear S-waves are unambiguously identified at all of the other seismic stations, such as IL06, IL08 and IL20, in Ilan Plain (Fig. 2c). A careful examination of S-wave frequency contents shows that the S-wave shadows observed at IL10 are not dependent on the frequency band (Fig. 5). For instance, S-wave arrivals are clearly identified at Station IL08 along the eastern coast of Taiwan at different frequency ranges (1–5 Hz, 0.5–1 Hz, 0.2–0.5 Hz and broadband), while it is hard to see S-wave arrivals at Station IL10 on Turtle Island. Those phenomena suggest that the S-wave shadows are only limited within the particular azimuth from the earthquake source (Fig. 2a). The same shadow zone is also found at the seismic stations on Turtle Island from some other earthquakes along a similar azimuth, such as Event 3 (Table 1) at the far distance (Fig. A4) and many other earthquakes in Fig. A2.

In addition to those earthquakes SE of Turtle Island (Fig. A2), the S-wave shadows are shown by the other crustal earthquake NE of Turtle Island (see Event 4 in Table 1 and Fig. 2a). In this case, S-waves are barely detected at Stations IL15 and IL19 or at stations on Turtle Island for Event 4. Thus, cross-checking both S-wave shadows from three earthquakes east of Turtle Island suggests a magma reservoir probably exists around the volcanic island, without consideration of the effect of earthquake depths at this moment.

## Ray-path Simulation

**Figure 6:**
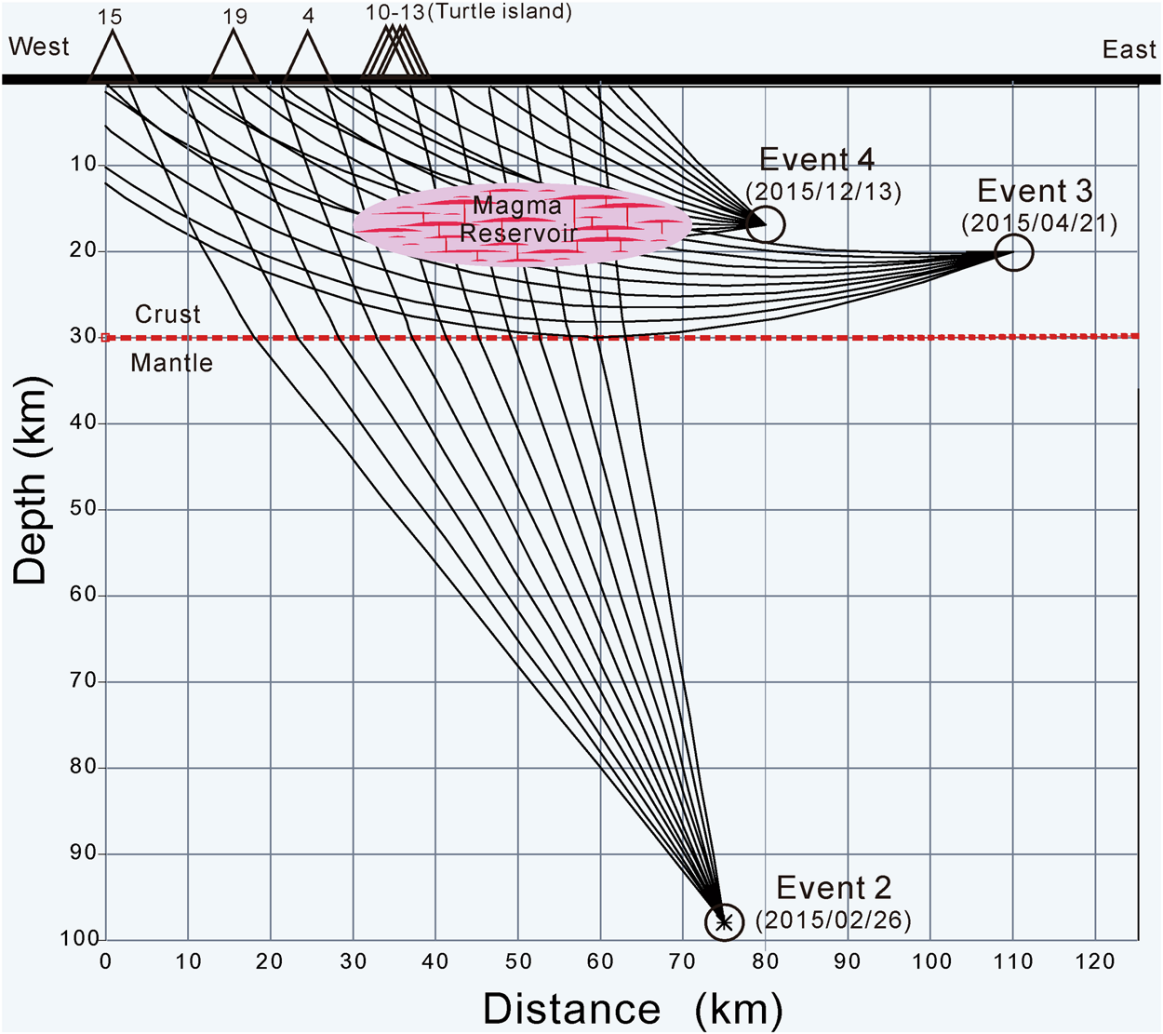
Schematic plot showing ray-paths of the S-wave shadows examined from three representative earthquakes (Events 2, 3, and 4 in Table 1). The magma reservoir is filled by molten sills and dikes in red and partial melting rocks in pink.

In order to estimate the possible location of the expected magma reservoir around Turtle Island from the S-wave shadows above, we employ a two-dimensional ray-tracing method^35^ for calculating ray-paths generated by three representative earthquakes at different depths and distances (Fig. 6). A simplified 2-D model of the mantle and overlying crust of ~30 km in thick is assumed for calculating ray-paths along the west-east profile. Although Turtle Island lies at the westernmost tip of the Okinawa trough, it is part of the volcanic arc based on the bathymetry and seismic data. The detail bathymetry shows Turtle Island is clearly connected to the whole sequence of the Ryukyu Arcs^36^. The subduction slab beneath Turtle Island is ~100 km, which is a typical depth beneath the volcanic arc in the Ryukyu subduction system^20,21,29^. In fact, the tomographic images^32,33^ show the Moho-depth offshore NE Taiwan is significantly larger than the typical thickness of 15–20 km in the Okinawa trough (Fig. 4). Therefore, it might be reasonable to assume the Moho-depth beneath Turtle Island is ~30 km. Finally, three different groups of direct S-wave ray-paths are simulated, including one group of nearly vertical waves from a deeper earthquake in the subduction zone and two groups of directed waves propagating through the crust from two shallower earthquakes at different epicenter distances.

The cross-check of three different groups of seismic rays shows the most likely location of the partial molten reservoir is roughly centered at the mid-crust (the depths between 16~18 km) beneath the Turtle Island area (Figs 3 and 4). The existence of such a partial molten reservoir provides a reasonable explanation for detecting not only S-wave shadows at Stations IL10–13 and IL04 from three earthquakes (Events 2, 3, and 4), but also those at Stations IL15 and IL19 from Event 4. Basically, the ray-paths generated by Events 3 and 4 provide some constraint for estimating the rough thickness of the partial molten reservoir while those generated by Event 2 and others in Fig. A2 roughly delineate the horizontal size of partial molten reservoir. Although the exact size and shape of the magma reservoir are not well defined by the limited available earthquake data here, a general geometry of the magma reservoir might be roughly delineated by combining with the LVZ from 3-D seismic tomography (Fig. 4). Thus, the estimated volume is approximately 10 km in both thickness and width, and more than 30 km in length with some uncertainties of ~3 km due to possible variations of earthquake locations, velocity models and ray-tracing methods.

## S-wave Reflection

**Figure 7:**
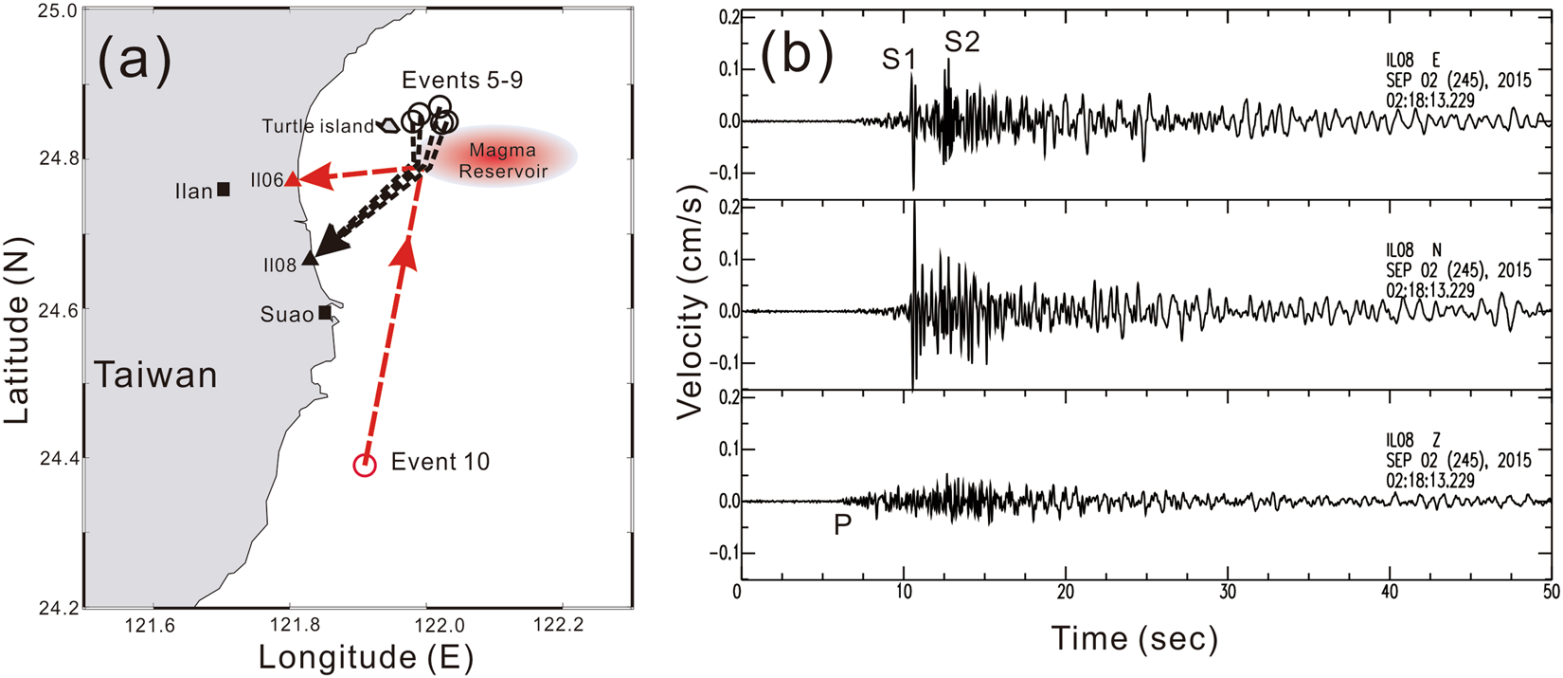
Schematic plot showing strong S-waves reflected from a magma reservoir. (**a**) Locations of 5 felt earthquakes (circles), seismic stations (triangles), a magma reservoir (red ellipse), and ray-paths (dashed-lines) in the Ilan area of Taiwan. (**b**) Three-component seismograms showing both S waves (S1 an d S2) recorded at Station IL08 and generated by Events 5–9.

In addition to the S-wave shadows, the detection of the melt-rich reservoir beneath Turtle Island is further confirmed by the strong S-wave reflections from local earthquakes. Careful examination of seismic data recorded in the Ilan seismic network shows strong S-wave reflections are clearly observed from 6 earthquakes (M > 4) in 2015 (Events 5–10 at Table 1). Among them, 5 earthquakes are clustered at the shallow crust (the depths <12 km) around 10–20 km eastward of Turtle Island (Fig. 7a). In addition to the direct S-waves, an unambiguous later S-waves are simultaneously recorded at Stations IL08, IL20, and IL18. For example, the 2^nd^ S-waves recorded at IL08 are consistently arriving around 2–3 sec after the direct S-waves (Fig. 7b). The comparable seismic amplitudes of both S-waves may imply that the 2^nd^ S-waves are strongly reflected from the melt-rich reservoir around Turtle Island. The comparable seismic amplitudes between the direct and reflected S-waves are associated with the radiation patterns of those earthquakes. The strong S-wave left the earthquake source around one of the fault planes in the focal mechanisms (Fig. A5), and thus we would expect to see comparable amplitudes between the direct and reflected S-waves in Fig. 6b.

Similarly, strong S-wave reflections are identified from another earthquake offshore the Suao area of eastern Taiwan on May 24, 2015 (Event 10 in Table 1). The 2^nd^ S-wave is recorded at Station IL06 with a delay time of ~3.68 s after the direct S-waves (Fig. A6).

Based on the travel-time delays between the direct and reflected S-waves (please see the details in the method section later), the heterogeneous melt-rich sills or dikes within the reservoir that is detected from the cross-check of S-wave shadows might be capable of reflecting the strong S-waves in different orientations and recorded at IL08 and IL06 (Fig. 6a). But there are still some uncertainties (~few kilometers) for estimating the exact geometry of the possible reflectors not only because the calculated travel-time delays are obtained from a simplified 1-D model here, but also earthquake locations have some uncertainties.

## Non-double-couple Earthquakes

**Figure 8:**
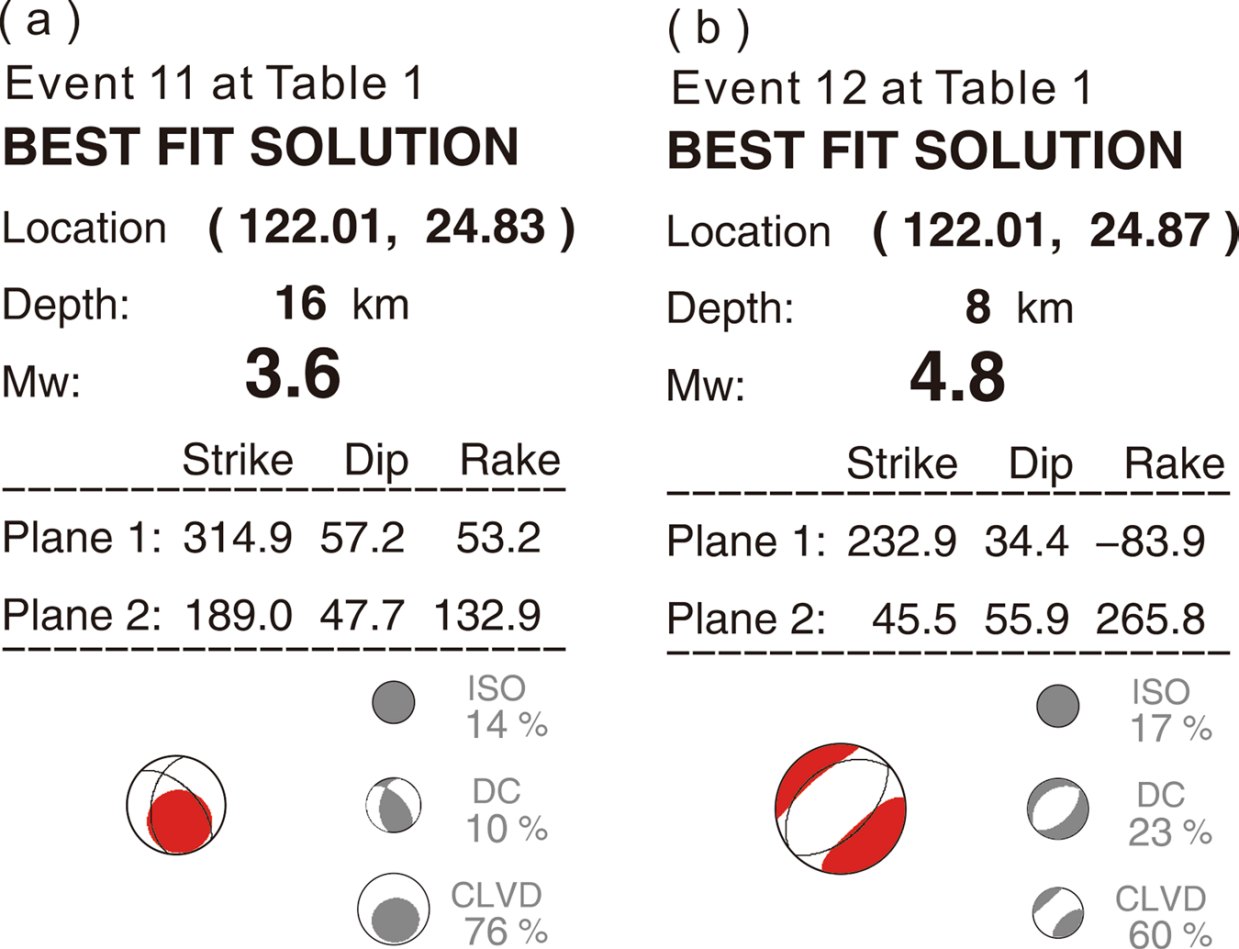
Moment tensor inversion results of two non-double-couple earthquakes (events 11 and 12 in Table 1) beneath Turtle Island. The moment tensors are decomposed into DC (double-couple), CLVD (Compensated Linear Vector Dipole) and ISO (Isotropic) components.

In order to know the possible activity of the magma reservoir beneath Turtle Island, we found some non-double-couple earthquakes with strong CLVD (Compensated Linear Vector Dipole) and ISO (Isotropic) from the real-time moment tensor reported by Institute of Earth Sciences, Academia Sinica (kttp://rmt.earth.sinica.edu.tw). The report has been routinely reported in real-time since 2014^37^, and it takes advantage of a grid-based moment tensor inversion technique and real-time broadband seismic recordings to automatically monitor earthquake activities in the vicinity of Taiwan. The centroid moment tensor inversion and a grid search scheme are applied to obtain the information of earthquake source parameters, including the event origin time, hypocentral location, moment magnitude and full moment tensor. All of these source parameters can be determined simultaneously within 117 seconds after the occurrence of an earthquake. The inversion procedure is based on a 3D grid system and 3D Green’s function database calculated by the Spectral-element method^38^ with a regional 3D velocity model^39^. We have occasionally found some non-double-couple earthquakes in and around Turtle Island since 2014. Among them, two of most representative earthquakes with significant CLVD and ISO components (Events 11 and 12 in Table 1) are shown at Fig. 8. The detailed waveform modeling and results are shown Fig. A11 and A12.

Both representative non-double-couple earthquakes with large ISO and CLVD components indicate some magma movements within the reservoir^40–42^. First, both earthquakes with large ISO components of 14% and 17% consistently indicate increasing of the source volume, such as an explosive mechanism. Second, the large vertical CLVD component (76%) at the depth of 16 km (Fig. 7a) further implies the vertical migration or rising of magmas within melt-rich dikes (Fig. 4a). The horizontal CLVD component (60%) at the depth of 8 km (Fig. 7b) probably suggests the horizontal migration of magmas within melt-rich sills (Fig. 4a). Magma rising within the magma reservoir might be directly driven by buoyance force due to higher temperature and lower density. The horizontal migration around the top of magma reservoir might be simply resulting from the limitation of the reservoir boundary. Certainly, those preliminary interpretations have to be confirmed by more high-resolution observations in the future.

## Melt-rich Sills and Dikes

Combining both observations of S-wave shadows and strong reflections in this study with general tectonics, geological data, background seismicity and the previous seismic images of 3D tomographic results, it is reasonable to expect a magma reservoir beneath Turtle Island. From the tectonic point of view, Turtle Island is not only situated at the westernmost end of the Okinawa trough but also along the Ryukyu volcanic arc (Fig. 1). Both tectonic features have strong volcanism in the Ryukyu subduction system^20,21,24,29,34,35^. The geological survey and analyses show Turtle Island is an active volcano^16,17,19,22^. The clustering earthquakes at the depth shallower than ~15 km in and around Turtle Island might be associated with the volcanic activities^26,29^. Two independent seismic images of tomographic results^32,33^ consistently show a reliable low-velocity zone beneath Turtle Island (Fig. 4). Finally, the non-double-couple earthquakes with strong CLVD and ISO components indicate the possible magma movements along dikes or sills within the magma reservoir beneath Turtle Island.

The S-wave shadows cross-checked by both nearly vertical and horizontal ray-path examinations suggest the molten magma (liquid material) is not on the top of the melt-rich reservoir alone, but probably heterogeneously distributed within the reservoir over a depth of several km in sill-like intrusions or melt-rich layers. The latter model could not be distinguished from the former model in the previous study^7^ because the magma reservoir was only identified using S-waves from deep earthquakes. In other words, previously there was no depth information to examine the magma reservoir from nearly vertical ray-paths. In addition to deep earthquakes, we anatomize the magma reservoir from the crustal earthquakes to distinguish the local distribution of molten magmas. As shown in Fig. 5, the missing S-waves at Stations IL04, IL15, and IL19 generated by the crustal earthquakes (Events 3 and 4) indicates that liquid material is heterogeneously distributed within the magma reservoir because the ray-paths propagate through the reservoir laterally.

The phenomenon of melt-rich sills and dikes heterogeneously distributed within the reservoir is also supported by strong S-wave reflections from different parts of the reservoir (Fig. 6a). Although the observations of S-wave shadows alone might be generated either by melt-rich volume or interfaces, we prefer the former rather than the latter based on the consistent observations of S-wave shadows and strong reflections. The major difference between them is that the melt-rich volumes would totally reflect S-waves while the interfaces might partially reflect S-waves and allow some seismic energy propagate through the reservoir. Thus, S-waves generated by shallow Events 5–9 at depths between 5 and 12 km are likely reflected from the uppermost portion of the reservoir, but the S-waves generated by Event 10 at a depth of 42 km may be reflected from its lateral portion. Events 5–9 are roughly located at the north of the magma reservoir while Event 10 is located at its south. Although the exact reflection points are currently not well defined, some strong reflectors might exist within the partial-molten reservoir in which many heterogeneous melt-rich sills or dikes with different orientations would be efficiently reflect strong S-waves from a variety of incidence angles. In other words, the reflection points for different earthquakes might not be the same, but they are probably close to each other within the reservoir.

Although the molten magma may be heterogeneously distributed within the magma storage volume, the total volume of the molten magma is expected to be very limited because the P-wave travel-time delay for waves propagated through the reservoir is too small to be consistent with a large volume of melt magma. Figure A7 shows an example of P-wave delays (~0.3 s) recorded at Stations IL10–13 on Turtle Island, in which the S-waves are missing. Similar results for small P-wave delays (~0.4 s) were reported from the detection of the magma reservoir beneath the Tatun volcano group in Taiwan^7^. Given a thickness of ~10 km for the partial melting reservoir beneath Turtle Island, the delay of ~0.3 s would be resulting from the low-velocity of 5.2 km/s within the reservoir that was ~19% lower than the assumed P-wave of 6.2 km/s in the mid crust (Fig. 4). Thus, the melt fractions of ~14% in partially molten rocks^43^. This result is slightly larger than that of 6–10% observed beneath Yellowstone hotspot^44,45^, but significantly smaller than the critical porosity of ~30% from petrological analyses at a Miocene volcano in southern Spain^43^. Consistent seismic observations of low melt porosity at different volcanic areas might simply reflect background incipient melting, while the high melt porosity regions are localized into heterogeneously distributed sills or dikes^43^. Therefore, instead of a large volume of molten magma, the small amount of P-wave delay is mainly the result of broadly partial melting rocks within the reservoir.

In fact, the interpretation of a magma reservoir beneath Turtle Island is also supported by the observation of non-double-couple earthquakes as well as the airborne geomagnetic survey. First, the large percentages (>60%) of CLVDs with some ISO component obtained from the moment tensor inversion (Fig. 8) strongly indicate those earthquakes were probably associated with magma movement within the reservoir. The vertical and horizontal CLVD components, respectively, suggest magma rising along dikes and migration along sills. Second, an airborne magnetic survey in and around Turtle Island shows several obvious high-magnetic anomalies^46,47^. The Curie depth estimated from 3D susceptibility model is ~5 km around Turtle Island, which is significantly shallower than those of 15–20 km beneath its surrounding areas such as the Ilan plain. Such an extremely shallow depth might reflect the magma reservoir beneath Turtle Island.

Based on the observations and interpretation above, we propose a hypothesis of a volcanic complex of melt-rich sills and dikes to describe the magma reservoir detected beneath the Turtle Island area, the Tatun volcano group, and probably many others in general. Like the presence of a succession of horizontal sills connected by vertical dikes beneath the Toba caldera^6^, the magma reservoir beneath Turtle Island may be composed of heterogeneous but limited melt-rich sills connected by vertical dikes within the partially molten rocks. In this model, the partial crystallization of basalt sills generates not only residual H_2_O-rich melts, but they can also provide heat and H_2_O for partial melting of pre-exiting rocks. One of the best exposed magma reservoirs filled with horizontal sills and vertical intrusions was found in the Torres Paine, Patagonia at the Argentina-Chile border^48^. Those horizontal sills were fed by vertical intrusions^49^. As a result, a concept model of the melt sill complex might be suitable for describing magma reservoirs not only beneath Turtle Island and the Tatun volcano group^7^ in Taiwan, but probably also other volcanoes in general.

In summary, consistent observations of both S-wave shadows and strong reflections recorded at seismic stations in the Ilan area of northeastern Taiwan clearly show a region of partial melt exists beneath Turtle Island. The magma reservoir not only reflects strong S-waves from the local earthquakes (Fig. 5a), but it also leaves some S-wave shadows from distant earthquakes (Fig. 2a). Although the exact geometry of the magma reservoir is presently not well constrained, its size and depth is roughly estimated from the limited seismic station, earthquake data and the LVZ of the previous seismic tomographic images. An ellipsoid-like body of ~30 km in length and ~10 km in both width and thickness might be expected at the mid-crust around a depth between 13 and 23 km with possible uncertainties of ~3 km due to variations of earthquake locations, velocity models and ray-tracing methods. The elongated direction approximately follows the strike of the Okinawa trough along the west-east direction in the northeast Taiwan area. This phenomenon indicates that the magma reservoir is probably developed by the back-arc opening in the Ryukyu subduction system. More detailed images will be obtained later after we deploy two dense seismic arrays from 2017 to 2020. One broadband seismic array (Formosa Array) with more than 140 stations will evenly cover the northern Taiwan area with the station spacing of ~5 km in average (Fig. A8). The other dense seismic array, with more than 600 portable 3-component geophones (Zland), will significantly improve the seismic images in the possible magma reservoirs in the Tatun volcano group as well as Turtle Island in the northern Taiwan area.

## Method

Reflection points estimated from the travel-time delay.

We have estimated possible reflection points according to the delay time between the direct (S1) and reflected (S2) measurements in the seismograms. Assuming a reasonable propagating S-wave velocity of ~3.2 km/s within a homogeneous space, the possible reflection points may be at any place on the surface of the ellipsoid body, with two focuses at the hypocenter and station. The size of the ellipsoid is determined by the delay time between the direct and reflected waves. For example, the possible reflection points on the ellipsoid for an earthquake (Event 5) recorded at Station IL08 is shown Fig. A6. To further constrain the possible reflection points, another ellipsoid body is calculated according to the strong S-wave reflection recorded at Station IL06 and generated by an earthquake (Event 10) offshore the Hualien area. The overlay points may be the best candidates as suitable locations to reflect the strong S-waves (Fig. A9). Since there are some uncertainties between observations and the assumed velocity model, we select the overlay points with a distance error of 1.5 km. As a result, all possible reflection points for generated 2^nd^ S-waves are shown in Fig. A10.

## Electronic supplementary material

Appendix Figures
