# Supplementary material for: Seismic Detection of a Magma Reservoir beneath Turtle Island of Taiwan by S-Wave Shadows and Reflections
Source: Sci Rep. 2018 Nov 6;8:16401. doi: 10.1038/s41598-018-34596-0 (PMC6219605; doi:10.1038/s41598-018-34596-0)
Supplement: Supplementary file 1 — Appendix Figures [file 41598_2018_34596_MOESM1_ESM.docx]

**Seismic Detection of a Magma Reservoir beneath Turtle Island of Taiwan by S-Wave Shadows and Reflections**

Cheng-Horng Lin ^1, 2, 3, 4, *^, Ya-Chuan Lai ^2, 3^, Min-Hung Shih ^1, 3^, Hsin-Chieh Pu ^5^ and Shiann-Jong Lee ^1^

1. Institute of Earth Sciences, Academia Sinica, Taipei, Taiwan
2. National Center for Research on Earthquake Engineering, National Applied Research Laboratories, Taipei, Taiwan
3. Taiwan Volcano Observatory at Tatun, Taipei, Taiwan
4. Dept. of Geosciences, National Taiwan University, Taipei, Taiwan
5. Central Weather Bureau, Taipei, Taiwan

(*) Corresponding author: Cheng-Horng Lin

P O Box 1-55, Nankang, Taipei, Taiwan

Email: lin@earth.sinica.edu.tw

Tel: 886-2-27839910 ext. 521

Fax: 886-2-27839159

Originally submitted to ***Scientific Reports*** on April 11, 2018

1^st^ revised on June 27, 2018

2^nd^ revised on August 17, 2018

3^rd^ revised on October 10, 2018

**Supplemental information (Appendix Figures):**


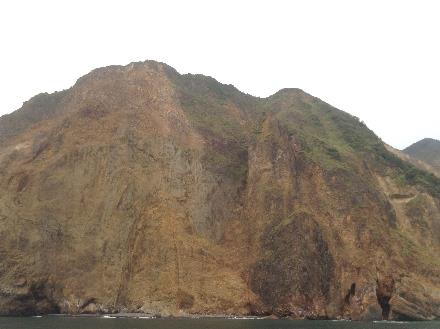

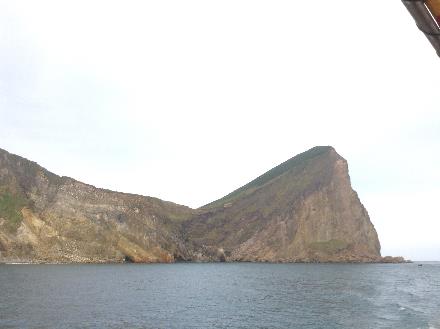


Fig. A1 Two photographs for showing the steep cliffs on Turtle Island of Taiwan.

**
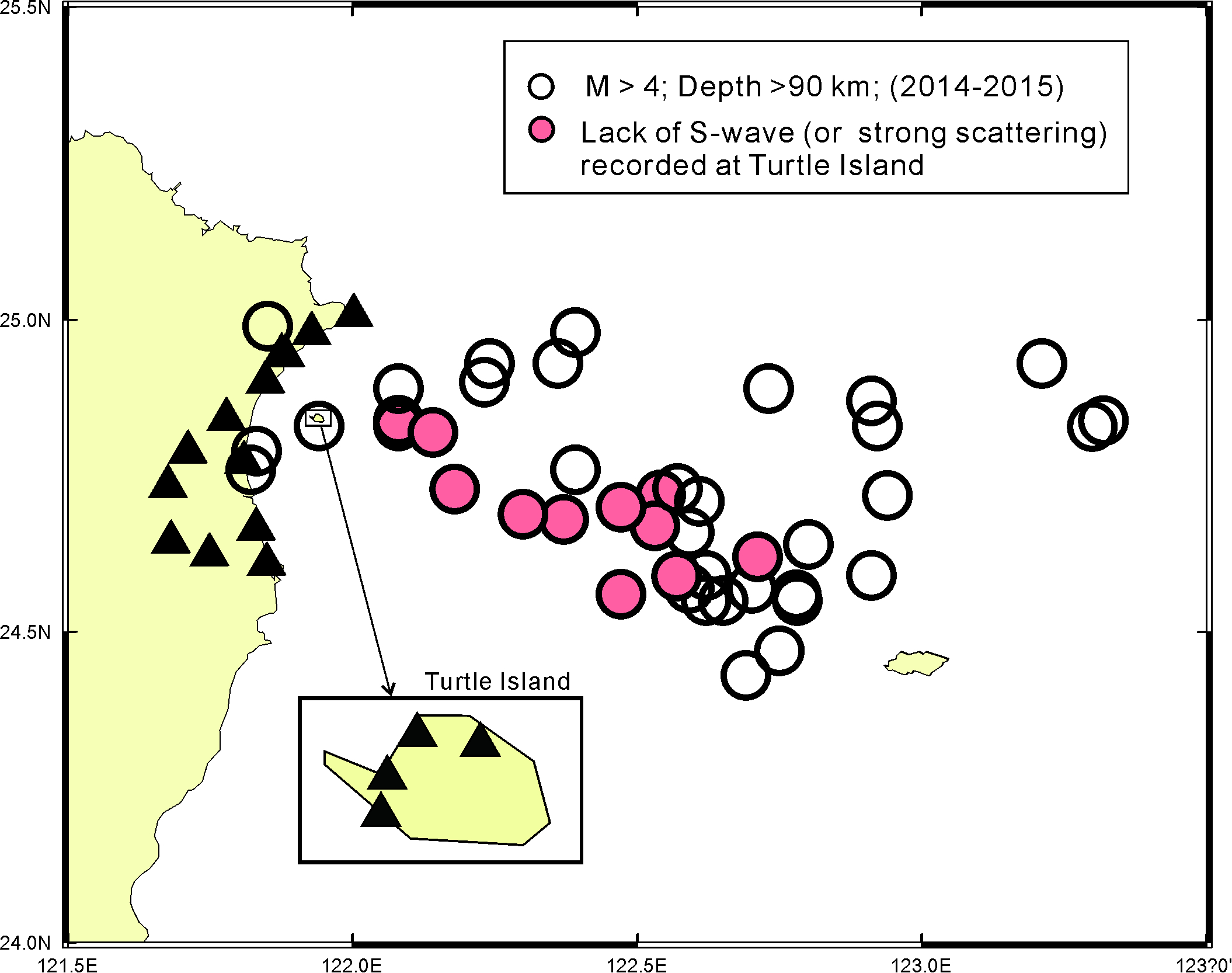
**

Fig. A2 Seismic stations (triangles) and deep earthquakes (circles) offshore NE Taiwan in 2014-2015. The lack of S-waves or strong scattering at Turtle Island are marked in red.


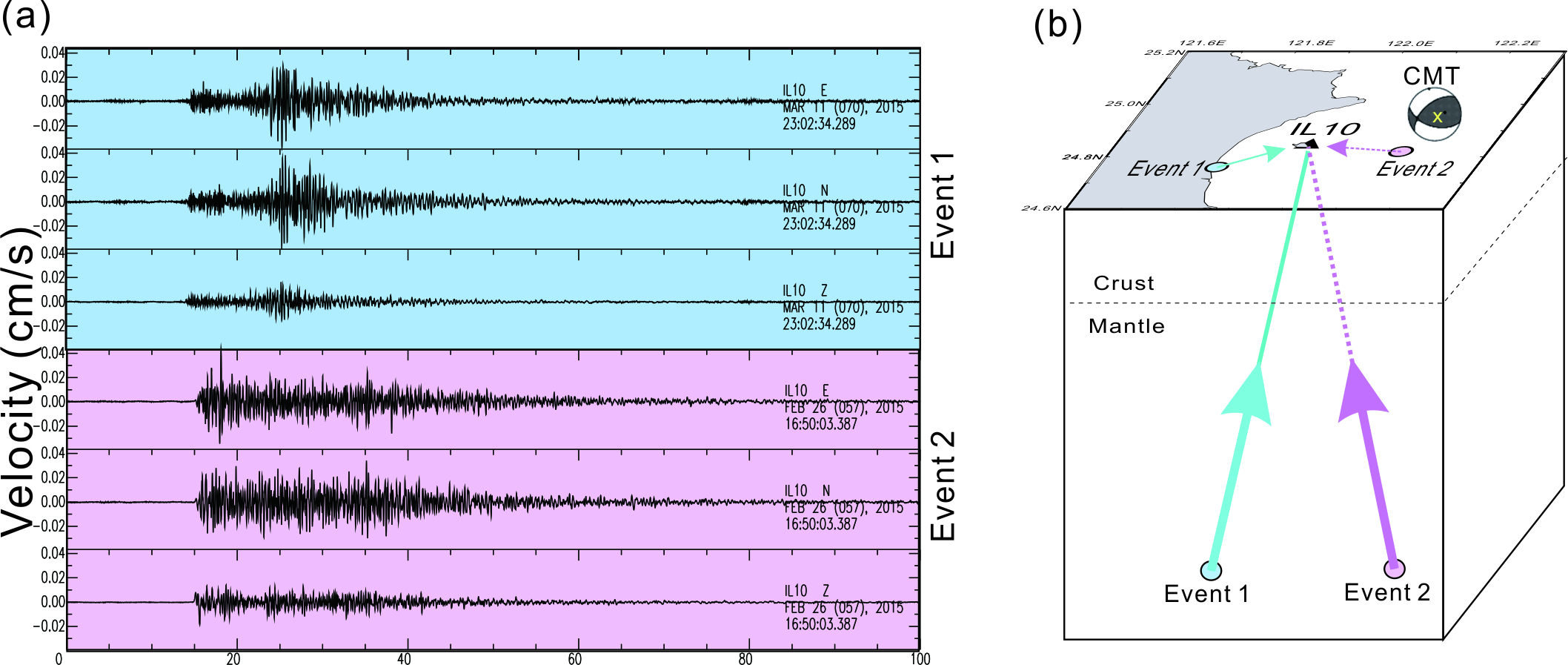


Fig. A3. Comparison seismograms generated from two deeper earthquakes. (a) Seismograms with and without S-waves recorded at IL10 from two earthquakes. (b) Locations of two earthquakes (Events 1 and 2) and seismic station (IL10) in the northeast Taiwan area. The incidence ray at Station IL10 is plotted by a yellow cross in the Centroid Moment Tensor (CMT) solution.


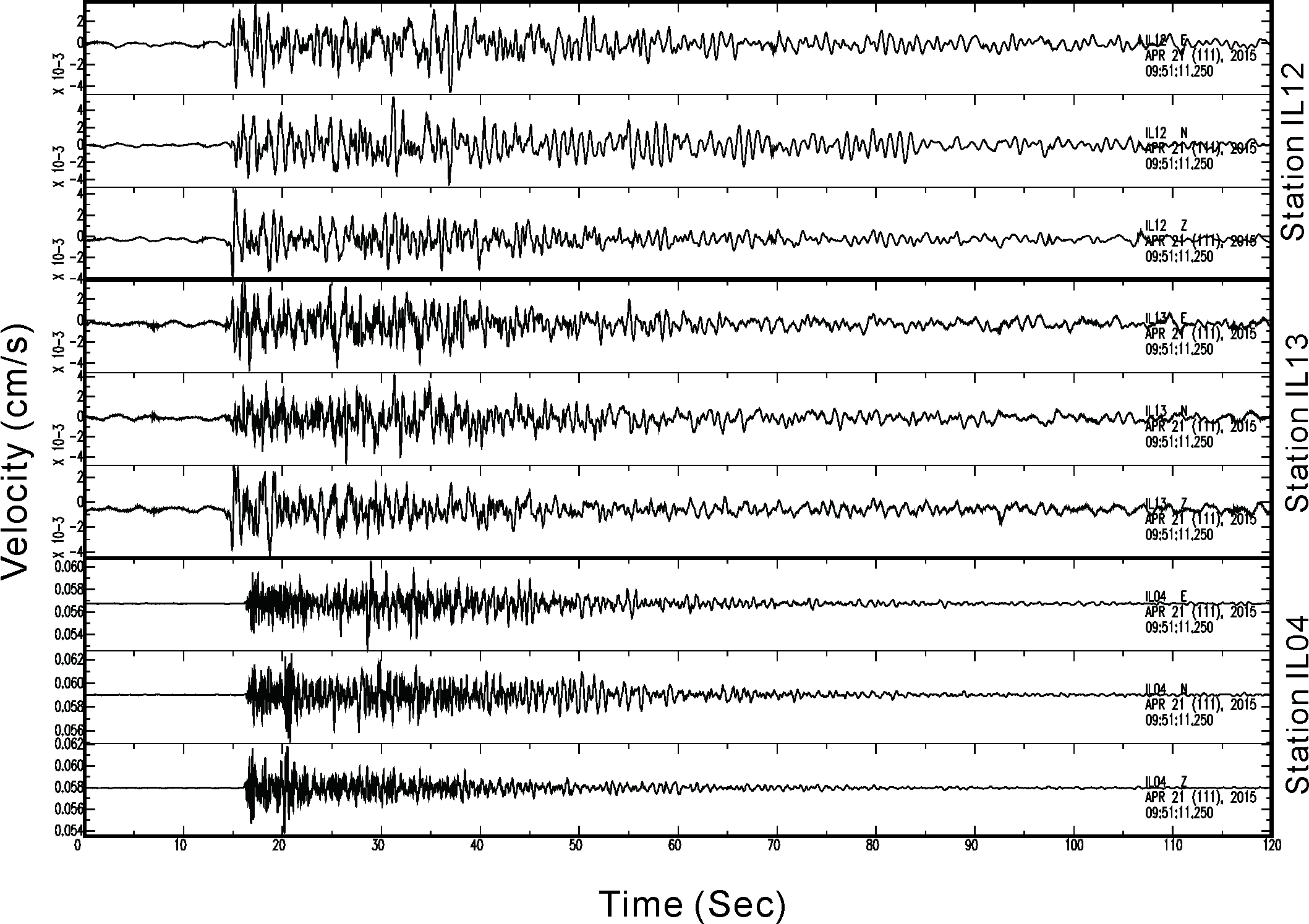


Fig. A4. S-waves were barely detected at Stations IL12, IL13, and IL04 from Event 3.


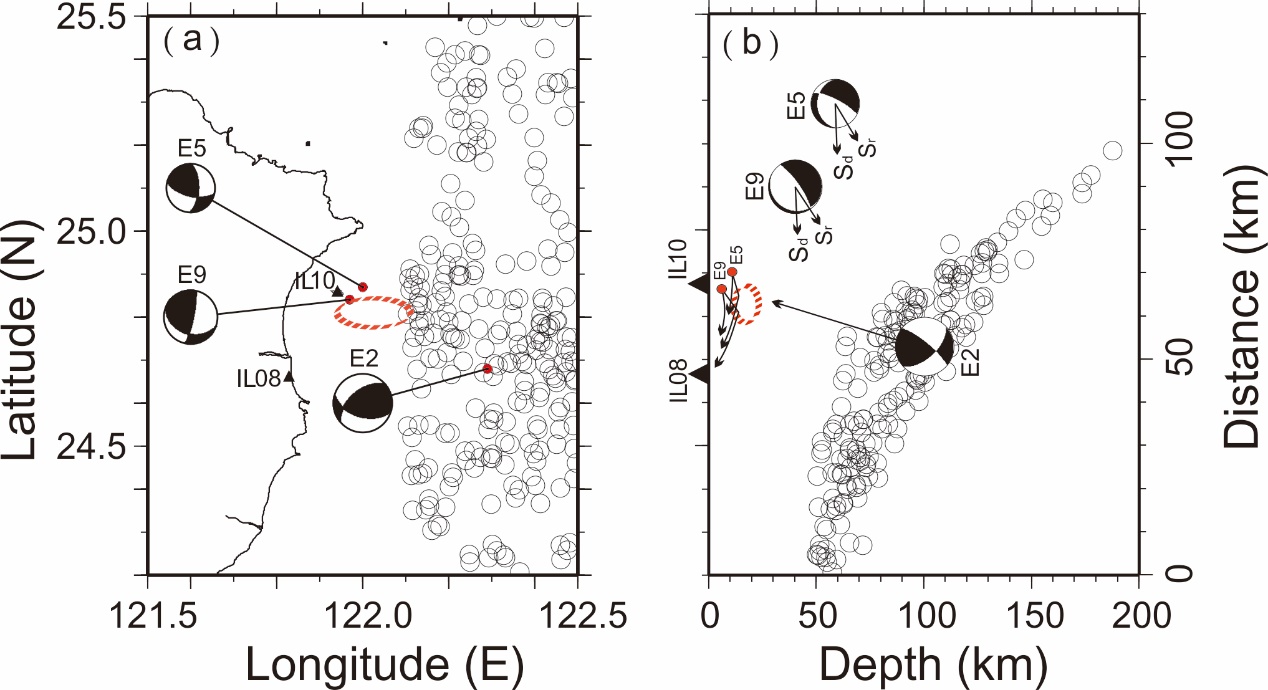


Fig. A5. Focal mechanisms projected in the low-hemisphere in the map view (a) and lateral view in the depth-profile (b) for showing radiation patterns from the earthquake sources. The direct and reflected S-waves left the source are roughly marked by Sd and Sr, respectively.


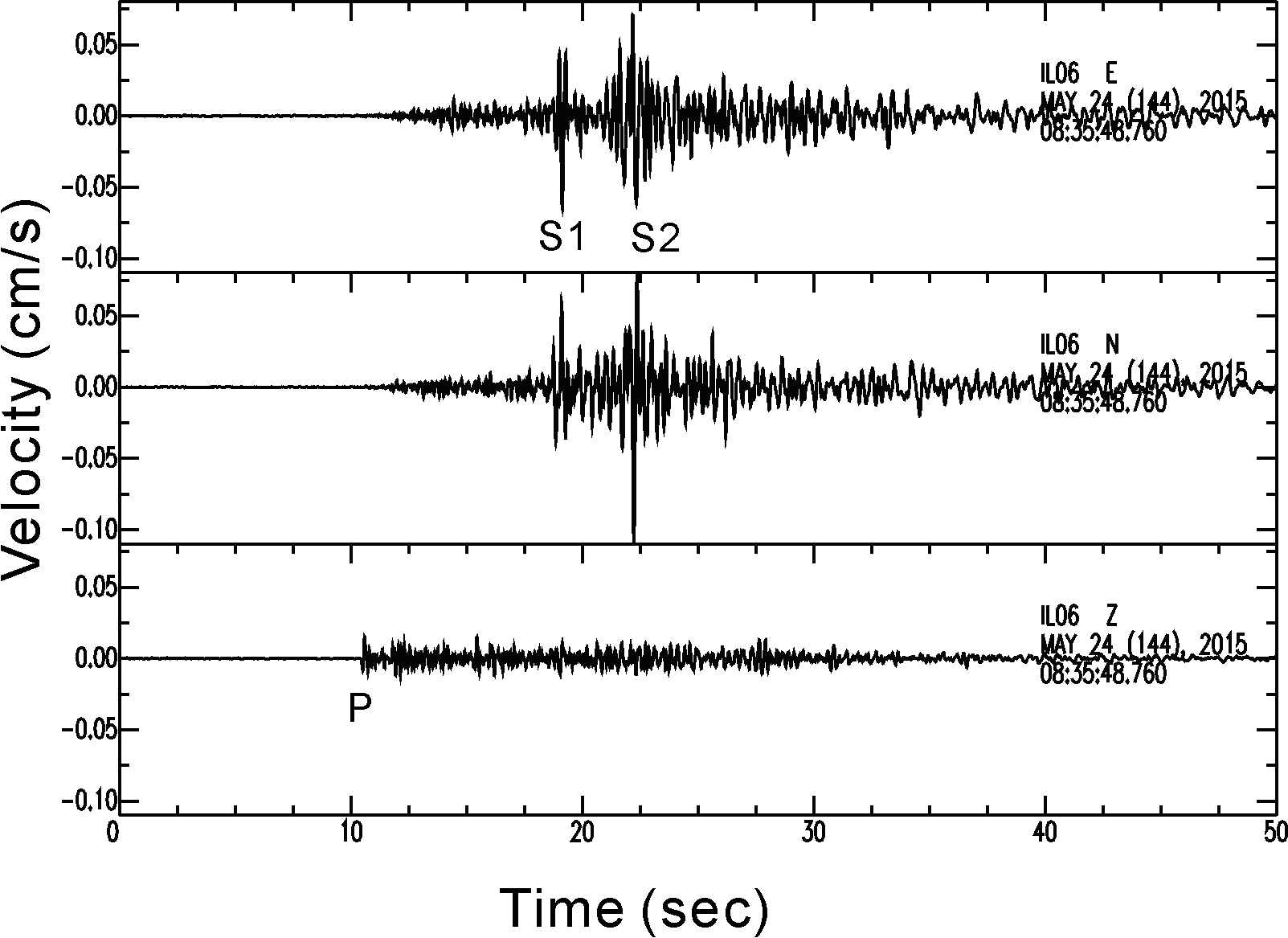


Fig. A6. Three-component seismograms showing the direct P and both S waves (S1 and S2) recorded at Station IL06 and generated by Event 10.


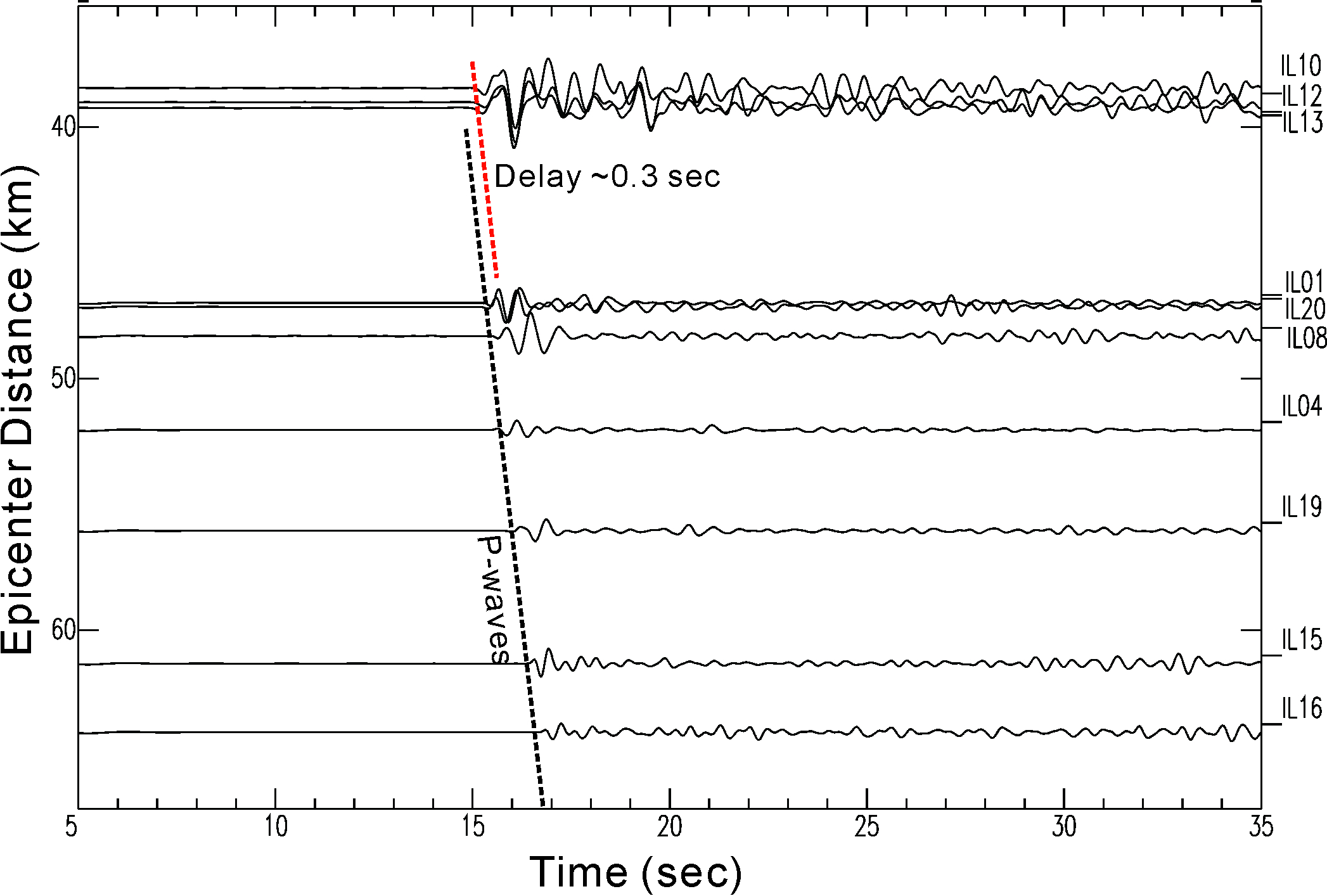


Fig. A7. Plots of P-wave arrivals with epicenter distances from Event 2. P-wave delays of ~0.3 s were recorded at Stations IL10-13 on Turtle Island.


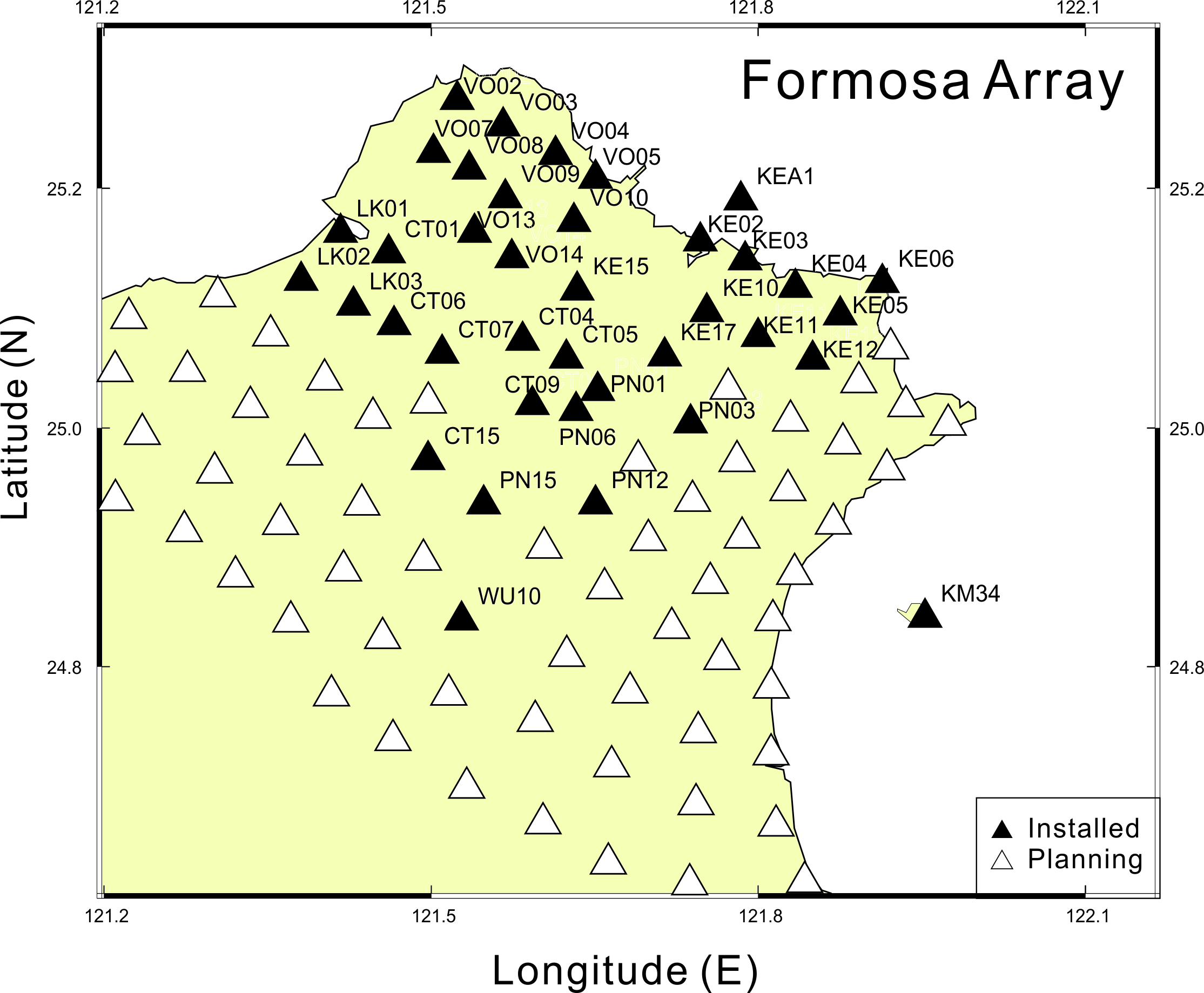


Fig. A8. Broadband seismic station installations in northern Taiwan with a station spacing of ~5 km. Sites shown by black triangles were successfully deployed in March 2018, and others (open triangles) will be installed in the following 3 years.


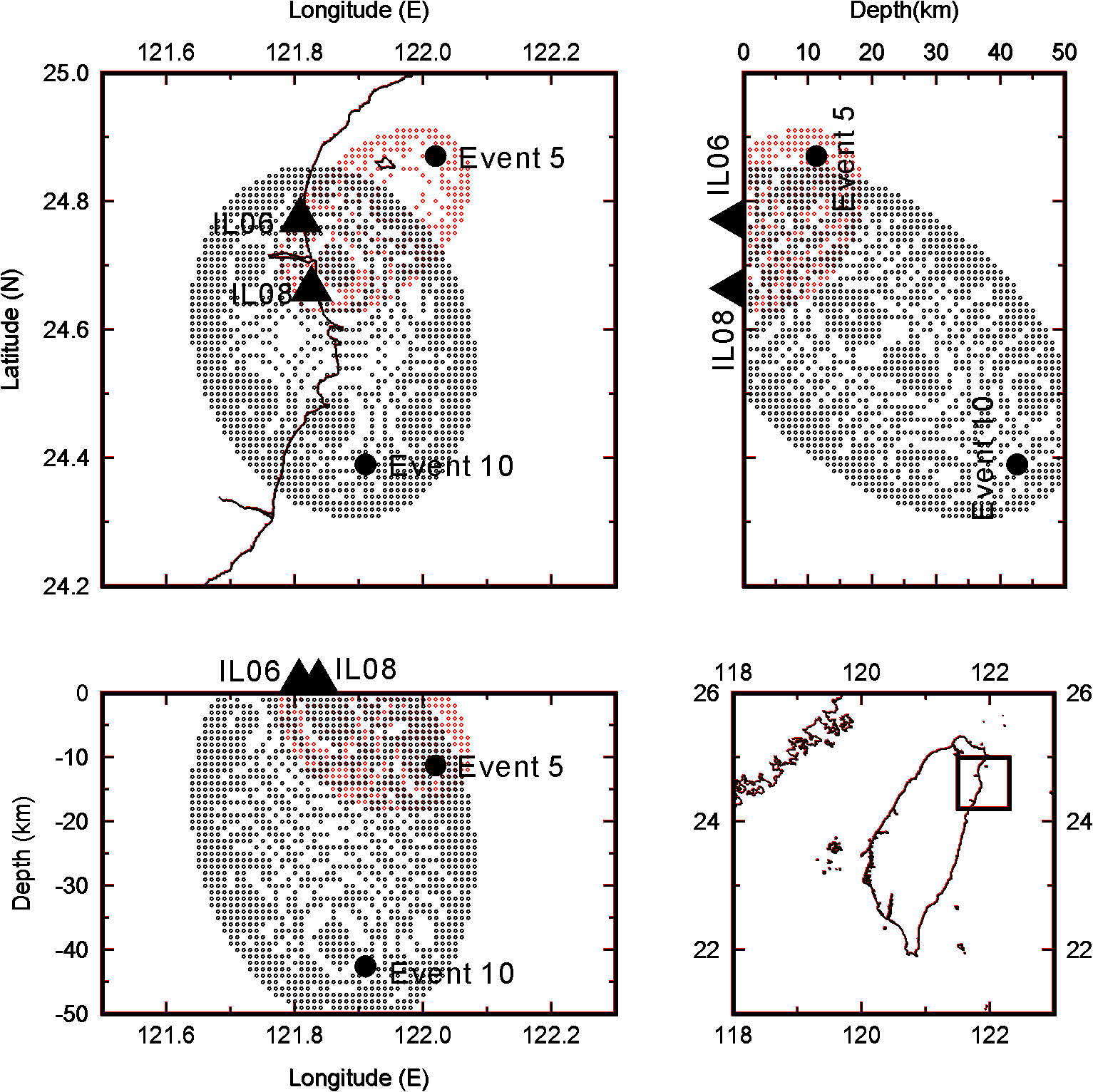


Fig. A9. Possible reflection points constrained from two earthquakes (Events 5 and 10). Three profiles for showing all possible reflection points on two ellipsoids.

**
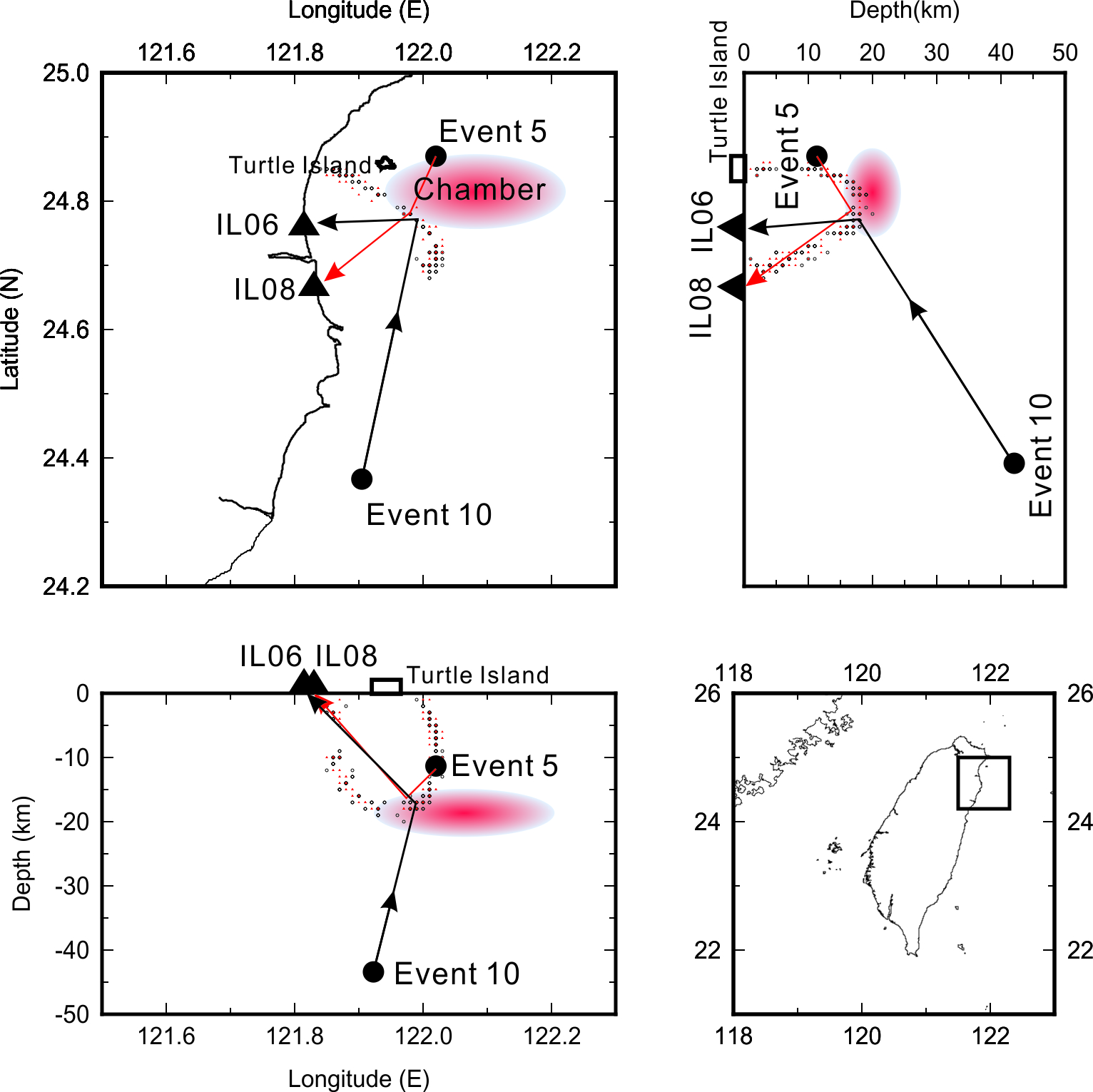
**

Fig. A10. Schematic projections of 3-D ray-paths reflected from a magma chamber marked in red. The small dots mark overlap points of possible reflections constrained by two earthquakes (Event 5 and 10) in Figure A6.


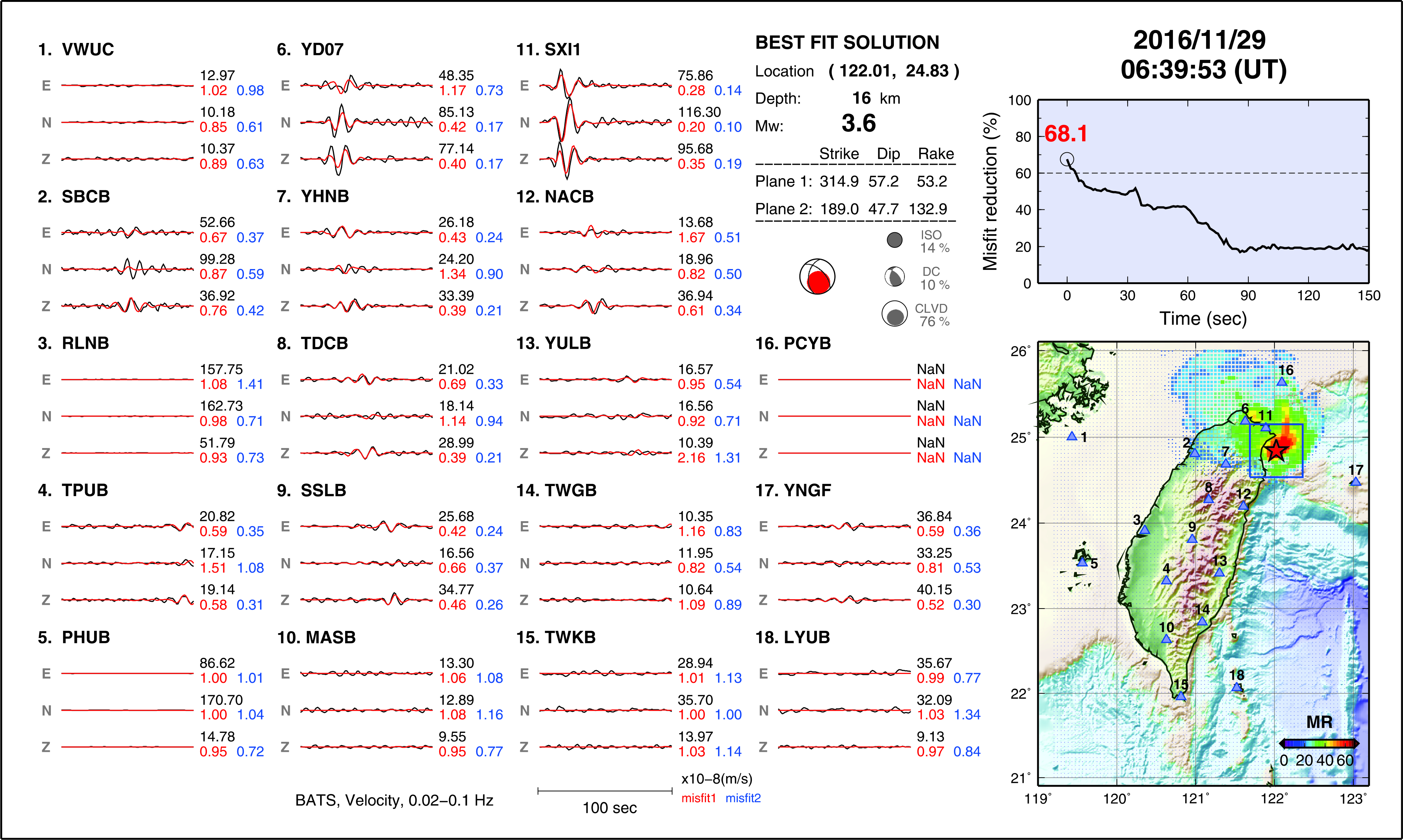


Fig. A11 Detailed results of moment tensor solution for Event 11 at Table 1.


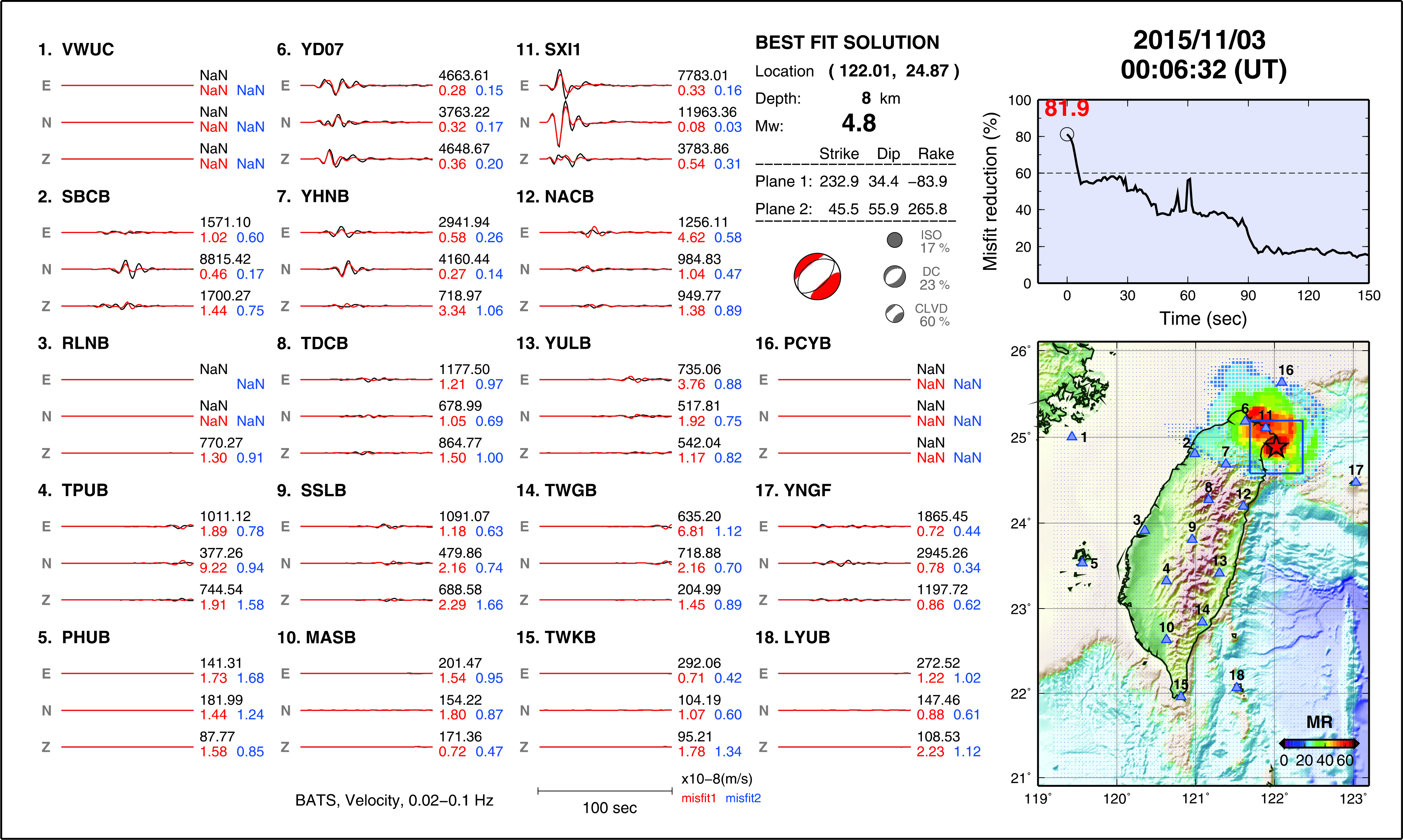


Fig. A12 Detailed results of moment tensor solution for Event 12 at Table 1.
